# Maternal immigrant status and survival among children with congenital heart disease

**DOI:** 10.23889/ijpds.v9i5.2545

**Published:** 2024-09-18

**Authors:** Qun (Grace) Miao, Sandra Dunn, Shi-Wu Wen, Jane Lougheed, Rebecca Griffiths, Carolina Lavin Venegas, Maria Velez, Mark Walker

**Affiliations:** 1 BORN Ontario; 2 OHRI; 3 CHEO; 4 ICES Queen's University; 5 Queen's University

## Objective

To examine associations between maternal immigrant status and survival of children with congenital heart disease (CHD).

## Approach

A retrospective population-based cohort study of hospital live births between April 2002 to September 2020 in Ontario, Canada was conducted at ICES. We identified children aged 0-17 years with a diagnosis of CHD from inpatient and health services datasets. The exposure was maternal immigrant status obtained from the IRCC permanent resident database. The outcome was time-to-death from after birth to <18 years olds. Multilevel Cox hazard regression models generated hazard ratios (HR) for associations between maternal immigrant status and children’s deaths while accounting for hospitals as a cluster factor and adjusting for maternal age at birth, neighbourhood income and education quantiles, comorbidities, a composite of severe maternal morbidity, gestational age at birth, birth weight, and infant sex. 

## Results

Relative to children born to non-immigrant mothers, the adjusted HR for death was 1.17 (95% CI:1.06-1.30) in children whose mothers were immigrants, and 1.33 (95% CI:1.07-1.65) in those from refugees. Moreover, compared to children residing in neighbourhoods with the highest income and educational levels, the adjusted HR of death for children who lived in neighbourhoods with the lowest income and educational level was 1.52 (95% CI: 1.24-1.88) and 1.47 (95% CI: 1.18-1.83) respectively.

## Conclusion

Children with CHD born to immigrant and refugee mothers, and those living in low socioeconomic status neighbourhoods had worse mortality. Health policy decision makers should target children with CHD from immigrant/refugee families and disadvantaged neighbourhoods to improve their survival.

